# Evaluation of the protective effect of aqueous-methanolic leaf extract of *Jatropha mollissima (Pohl.) Baill*. on doxorubicin-induced cardiotoxicity in rats via modulating inflammatory markers and oxidative stress

**DOI:** 10.3389/fphar.2025.1634378

**Published:** 2025-09-08

**Authors:** Muhammad Omer Iqbal, Qianqian Wang, Majid Manzoor, Imran Ahmad Khan, Yuchao Gu, Jin Chen, Xiao Wu

**Affiliations:** ^1^ College of Biological Engineering, Qingdao University of Science and Technology, Qingdao, China; ^2^ Key Laboratory of Marine Drugs, the Ministry of Education, School of Medicine and Pharmacy, Ocean University of China, Qingdao, China; ^3^ Fatima Tu Zahara Department of Life Sciences, Muhammad Institute of Medical and Allied Sciences, Multan, Pakistan; ^4^ Institute of Drug Discovery Technology, Ningbo University, Ningbo, China; ^5^ Qian Xuesen Collaborative Research Center of Astrochemistry and Space Life Sciences, Ningbo University, Ningbo, China; ^6^ Department of Pharmacy, MNS University of Agriculture, Multan, Pakistan; ^7^ Qingdao Central Hospital, University of Health and Rehabilitation Sciences (Qingdao Central Medical Group), Qingdao, China

**Keywords:** cardioprotection, doxorubicin, CVds, anticoagulant, fibrosis, oxidative stress, inflammation

## Abstract

**Introduction:**

Jatropha mollissima (Pohl.) Baill is a traditional medicinal plant reputed for its hepatoprotective and nephroprotective properties. However, its potential cardioprotective and anti-inflammatory effects, both *in vitro* and *in vivo*, remain underexplored.

**Aim of the study:**

This study conducted a series of *in vitro*, *in vivo*, and *ex vivo* experiments to determine the cardioprotective properties and anti-inflammatory effect of the aqueous-methanolic leaf extract of *J. mollissima*. Doxorubicin-induced cardiotoxicity, thrombolytic, anticoagulant, antioxidant, vasorelaxant, anti-inflammatory, and calcium channel-blocking activities were determined.

**Materials and Methods:**

The study involves a phytochemical evaluation, along with HPLC analysis. The antioxidant activities of the *J. mollissima* extract were determined using *in vitro* assays, including DPPH, SOD, NO, and H_2_O_2_. *In vitro* and *in vivo* anticoagulants, antithrombolytic agents, vasorelaxants, and biochemical assays were performed to determine Jm’s protective effect. Cardiac inflammatory markers (TNF-α, IL-1β, IL-6, and IL-10) were evaluated via real-time PCR. Doxorubicin was used as the positive control.

**Results:**

In an in-vitro anticoagulant experiment, *J. mollissima* displayed a substantial increase in activated partial thromboplastin, prothrombin, and clotting time in a dose-dependent manner (20%, 10%, and 5% dilutions) compared with heparin (250 IU/mg) and distilled water. While in-vivo anticoagulant experiment showed a substantial increment in clotting time, prothrombin time, bleeding time, and activated partial thromboplastin time in a dose-dependent manner (25 mg/kg, 50 mg/kg, and 100 mg/kg) in rats after 1-week of treatment in comparison with heparin (50 IU/mg) and distilled water. For the thrombolytic (*in vivo* and *in vitro*) experiments, dose-dependent (20%, 10%, and 5% dilutions) significant (p < 0.05) clot lysis was observed compared to streptokinase (30,000 IU) and distilled water. For antioxidant activity, doxorubicin (intraperitoneally at 10 mg/kg at 0 days) was given, blood samples were extracted (at 21st day) to determine cardiac damage by measuring DPPH, SOD, NO, CK-MB, LDH, Troponin I, serum sodium, and serum potassium in which aqueous-methanolic extract in a dose-dependent manner (600 and 400 mg/kg dilutions) displayed significant (p < 0.005–0.000) decrease in serum level. The cardiac weight-to-body weight ratio showed significant resistance to necrosis caused by the doxorubicin-induced toxic group. HPLC analysis revealed the presence of gallic acid, mandelic acid, quercetin, pyrogallol, and rutin. Gene expression analysis revealed that Jm reduced proinflammatory cytokines (TNF-α, IL-1β, and IL-6) and upregulated the anti-inflammatory cytokine IL-10, with effects comparable to those of doxorubicin.

**Conclusion:**

Thus, the anticoagulant, antioxidant, cardioprotective, anti-inflammatory, and thrombolytic properties of *J. mollissima* are attributed to the presence of various phytochemical constituents, which may act on multiple factors. Its beneficial actions are attributed to the modulation of oxidative stress and neuroinflammatory pathways, suggesting its therapeutic potential in managing cardiotoxicity and other complications.

## 1 Introduction

Cardiovascular diseases (CVDs) are heart or blood vessel abnormalities. Stroke and abrupt death can result (Karlsson et al., 2001). In many affluent countries, CVDs have been the leading cause of disability, death, and morbidity for years. Emerging nations are responding to this pandemic at alarming rates (Members et al., 2012). Many feel that vascular abnormalities cause coronary artery disease and cerebrovascular disease, which contribute to arrhythmias and heart attacks (Khan et al., 2015). Dickneite et al. (1995) say thrombus development kills most people in wealthy countries. Coronary artery problems, DVT, heart attacks, strokes, and pulmonary embolism can result from thrombosis. Thromboembolic illnesses can result from arteriosclerosis, infection, or trauma, which slow blood flow and cause clots.

The anthracycline antibiotic doxorubicin treats breast, lung, stomach, ovarian, and childhood cancers, as well as hematologic malignancies, neuroblastomas, thyroid, bronchogenic carcinoma, and Wilms’ tumor (Kirstein, 2009). The side effects of anthracyclines limit its use. Multiple pathways may produce its toxicity (Manzoor et al., 2022). Doxorubicin was initially produced in the 1960s by *Streptomyces* peucetius. Blocking topoisomerase II, which increases cleavable enzyme-DNA-linked complexes during DNA replication, prevents polynucleotide strand ligation after double-strand breaks.

Doxorubicin induces cardiotoxicity through various mechanisms, including the generation of reactive oxygen species (ROS) from oxidative stress mediated by Cytochrome P450 reductase, which converts doxorubicin’s quinone form into the free radical semiquinone form in mitochondrial cells and then into superoxide free radicals via ferrous ions (Cejas et al., 2023). Adenylate cyclase activity affects cardiac cell ATP generation and transmission (Cejas et al., 2023). Doxorubicin induces hypercoagulation by elevating expression of tissue factor (TF) and activated thrombin generation (Grover et al., 2021; Manzoor et al., 2022). Doxorubicin increases NO levels and endothelial NOS (eNOS) and protein activity, generating superoxide. Endothelial ROS apoptosis, calcium overload in the ventricular myocardium, lysosomal changes, changes in the Na+/K + ATPase pump, and the Ca^2+^ ATPase pump all contribute to cardiotoxicity (Ruggeri et al., 2018). Iron-dependent oxidative damage in myocytes is caused by semiquinone metabolites. Hydrogen peroxide is produced when these metabolites delocalize ferritin iron (II). Continued processing produces hydroxyl radicals and lipid peroxidation (Feng et al., 2022).

The heart is more susceptible to doxorubicin’s free radical-producing actions than the kidneys because it lacks antioxidants such glutathione, superoxide dismutase, and catalase. Cardiolipin in the heart makes it more susceptible than the kidneys and liver. Doxorubicin-affinity mitochondrial membrane phospholipids are stored in cardiac cells (Yousefi et al., 2023). A tissue plasminogen activator effectively dissolved the clot (Hall, 2016). Modifiable risk factor medication therapy reduces CVD events significantly. Clinical practises with HMG-CoA reductase inhibitors reduce coronary disease events (Roderick et al., 2005). Besides pharmacological medications, medicinal herbs including angina pectoris, arrhythmia, systolic hypertension, congestive heart failure, and venous insufficiency are used to treat and minimize CVD risks (Sawant and Godghate, 2013). Although antioxidants are the main emphasis, some plant metabolites may potentially be thrombolytic and anticoagulant (Gilani et al., 2010).

Target tissues determine the names of creatine kinases: CK-BB for the brain, CK-MB for the heart, and CK-form for the muscles. The heart is more sensitive to reactive oxygen species when glutathione, superoxide dismutase, and catalase levels are low. Doxorubicin strongly binds to cardiolipin in cardiac cell mitochondria (Khan et al., 2022). Lactate dehydrogenase has two isoforms, the H form for the heart and the M form for muscles. Both are indicators for heart muscle injury. LDH elevation indicates heart damage. It quantifies cell health (Khan et al., 2021a). To detect cardiac damage, troponin is sensitive. Troponins I, C, and T line the muscle filament (Khan et al., 2021b). Electrolyte profiles monitored acid-base imbalances and therapy efficacy. Electrolytes like sodium, potassium, chloride, and bicarbonate can help diagnose medical issues (Muehlberg et al., 2023; Hinchcliff et al., 2013).

Cardiac Inflammation is a crucial contributor to cardiac dysfunctions, particularly in cardiotoxixity and related disorders (Rom et al., 2019). Elevated levels of proinflammatory cytokines such as interleukin-6 (IL-6), interleukin-1 beta (IL-1β), tumor necrosis factor-alpha (TNF-α), and dysregulation of interleukin-10 (IL-10) an anti-inflammatory cytokine play a crucial role in cardiac impairment (Bourgognon and Cavanagh, 2020; Olajide et al., 2013; Porro et al., 2020). TNF-α disrupts the blood-brain barrier, induces cardiac apoptosis, and impairs synaptic plasticity to cause cardiac deficits. In addition, IL-1β worsens cardiac inflammation by hindering hippocampal neurogenesis, and IL-6 alters cardiotransmitter metabolism to promote cardioinflammatory pathways. Conversely, IL-10 is protective by counteracting TNF-α and IL-6 and maintaining neuroimmune balance. However, reduced IL-10 levels in cardiotoxicity lead to unchecked inflammation, increasing the risk of cardiac failure. Research on natural products for cardiac complications is central due to their potential to provide safer and more cost-effective therapeutic alternatives (Jugran et al., 2021). Unlike synthetic drugs, natural compounds often exhibit fewer adverse effects, making them more suitable for long-term use. They also offer multi-targeted mechanisms to address cardiac and metabolic dysfunction, oxidative stress, and inflammation simultaneously. Moreover, numerous studies using various animal models have investigated the medicinal effects of various plants in controlling cardiotoxicity and its related complications. (Jugran et al., 2021).

*Jatropha mollissima* (Pohl.) Baill. belongs to the family Euphorbiaceae and is native to southern China, as well as parts of East and Southeast Asia. In traditional Chinese medicine (TCM), it is widely used for different activities, mainly as an anti-inflammatory (Iqbal and Yahya, 2021; Iqbal et al., 2022), antivenom (Abogmaza et al., 2020; Bastos-Cavalcante et al., 2020), nephroprotective (Iqbal et al., 2021a), wound healing (Khan et al., 2020), veterinary vermifuge (de Queiroz Neto et al., 2019), antioxidant (Iqbal et al., 2021b; Iqbal et al., 2022), anthelmintic, antimicrobial (Iqbal et al., 2022), antibiotic modifying, and antibacterial activities (Iqbal et al., 2021a).

Phytochemical analysis of *J. mollissima* leaf extract showed the presence of orientin, vitexin, isovitexin, isoschaftoside, schaftoside, vitexin-2-O-rhamnoside, luteolin, apigenin, luteolin-7-O-glucoside, spinasterol, n-triacontane, β-sitosterol, catechin, chlorogenic acid, ellagic acid, ferullic acid, gallic acid, cinamic acid, methyle gallate, syringic acid and quercitin (Iqbal and Yahya, 2021; Hsu and Yen, 2008).

Previously aqueous-methanolic leaf extract of *J. mollissima* reported for its antioxidant, antitumor, hepatoprotective, antiobesogenic, antidiabetic, antihypertensive, and antihyperlipidemic effects which drove us to evaluate its cardioprotective potential. The aqueous-methanolic leaf extract of *J. mollissima* was tested for thrombolytic, anticoagulant, antioxidant, and HPLC/phytochemical screening properties.

## 2 Materials and methods

### 2.1 Plant material collection

*J. mollissima* leaves were gathered from the Muhammad Institute of Medical and Allied Sciences garden in Multan, South Punjab. A highly skilled taxonomist from Bahauddin Zakariya University helped with the plant identification. Multan and herbarium specimens were submitted for the record (R. R. Stewart F. W. Pak. 707/12).

### 2.2 Preparation of plant material

We let the newly harvested plant leaves dry in the shade. After removing any dirt or debris, the dried leaves were ground to a fine powder using a specialized herb grinder. The powdered plant material was preserved in an airtight container. The powdered material was extracted using a maceration operation in a aqueous-methanolic (30:70) mixture, following a conventional method described (Khan et al., 2015). At 37 °C and low pressure, the crude extract was evaporated using rotatory evaporator. Put in a lab fridge at −2° in sealed containers (Ain et al., 2022). The formula was used to determine an approximate 10% extract yield.

$$\%\text{ yield}=\left(\text{weight after evaporation }/\text{ dry weight of leaves}\right)\times 100$$

### 2.3 Animals

By relevant ARRIVE guidelines (http://arriveguidelines.org) and following the guidelines of the National Institutes of Health for the Care and Use of laboratory animals, Male Wistar Albino rats ranging in weight from 250 to 330 g (8–10 weeks of age) were procured from the animal house of Multan’s Muhammad Institute of Medical and Allied Sciences. Polycarbonate cages housed the animals in a typical laboratory environment (12 h light/dark cycle, 22 °C ± 2 °C, 50% ± 10% humidity). The dust was changed every 3 days. Water and standard food pallets were made available to them at all times.

### 2.4 Ethical approval

The experiments were conducted by the guidelines of the National Institutes of Health and the Helsinki Ethical Code (Council, 2010). The Animal Ethical Committee (AEC) of the Muhammad Institute of Medical and Allied Sciences approved the project (MIMAS/07–24/2631). *In vitro* experiments on human blood was also approved (MIMAS/07–24/2631/1).

### 2.5 Chemicals and reagents

We bought streptokinase from Pakistan’s Highnoon Laboratories Ltd. Mehran Traders Ltd. of Pakistan was the source for the heparin vials. Doxorubicin, formalin, and ketamine were purchased from Sigma Aldrich (Germany). Xylazine was purchased from Prix Pharmaceutical Lahore. The following reagents were acquired from Javaid Pharmaceuticals Ltd. Pakistan: Meyer’s reagent, Folin-Ciocalteu reagent, PT, and APTT reagents. Merck, Germany, supplied the methanol, hydrochloric acid, sodium hydroxide, and H_2_SO_4_. British company BDH Laboratory Pvt. Ltd. supplied the FeCl_3_. DPPH and NADH were purchased from Sigma-Aldrich.

### 2.6 Preliminary phytochemical screening

The aqueous-methanolic leaves extract of *J. mollissima* was subjected to phytochemical screening in accordance with established protocols to identify potential constituents of key phytochemical classes, including alkaloids, anthracene derivatives, anthraquinones, anthocyanins, phenolic metabolites, coumarins, saponins, triterpenes, and tannins (Ain et al., 2023).

### 2.7 HPLC analysis

Khan et al. (2022) conducted HPLC analysis to quantify phenolic acids and polyphenolic metabolitess, adhering to USP and ICH requirements. The wavelength employed for poly. The identification of phenols occurred at 280 nm, while the furnace column temperature was set at 35 °C. The Ultimate 3,000 liquid chromatography system, featuring a 5 cm flow cell DAD and Chromeleon system administration, was utilized for HPLC analytics. The reversed-phase Acclaim C18 column (5-micron particle size, 250 mm × 4.6 mm) was employed for differentiation. A total of 30 mg of dry methanol and water extract was separately dissolved in 25 mm of mobile phase solvent. The 0.45 µm membrane filter processed the sample solution prior to injection (methanol: 0.5% acetic acid in water: 1.9) into the HPLC apparatus. The high-performance liquid chromatography analysis was performed utilizing a methanol phase with mobile solvent (Solvent A) and acetic acid solutions (Solvent B), with a duration of 105 min allocated for each sample. The HPLC spectrum library documented and preserved each standard. The identification criteria for Alhagi camelorum chemicals were established by comparing the retention time and spectra of unknown metabolitess to the HPLC standard library. In the extracts, phenolic acids and flavonoids were quantified using a calibration graph by plotting spikes against the corresponding standard control sample. The statistics have been presented as mean ± standard error from three independent evaluations.

### 2.8 *In-vitro* experiments on human blood

#### 2.8.1 Anticoagulant activity, volunteers and selection conditions

Five distinct test tubes were used to transfer 3 mL of blood collected from 25 healthy human volunteers who were not using any form of hormonal or pain medication from last 2 weeks. The five test tubes were filled with 0.2 mL each of aqueous-methanolic extract (20%, 10%, and 5% dilutions), distilled water, and heparin (250 IU/mg) and incubated at 37 °C. Then, a stopwatch was used to measure the clotting time (CT) (Ain et al., 2022; Farooq et al., 2023; Mulloy et al., 2016; Hussain et al., 2014; Furlanello et al., 2006).

#### 2.8.2 Analysis of activated partial thromboplastin time (APTT) and prothrombin time (PT)

Sodium citrate-containing tubes were centrifuged for 5 min at 3,000 rpm with blood samples (3 mL) transferred from 25 healthy human volunteers. Plasma was transferred to each participant’s unique eppendorf tube using micropipettes. In separate eppendorf tubes for each participant, the same volume of plasma was combined with varying concentrations of aqueous-methanolic extract (5%, 20%, 10%, and 5% dilutions; 100 μL), heparin (100 μL, 250 IU/mg), and distilled water. The sample was incubated at 37 °C for 5 min to find the PT (Alesci et al., 2009). The Eppendorf tube was then combined with 200 mL of PT reagent for each test, and the time was recorded as PT using a stopwatch. To find the APTT, the plasma sample is combined with 100 L of APTT reagent. After a minute of incubation, 100 mL of calcium chloride was added and left to clot for 15 s; the clotting time, as determined by APTT with a stopwatch, was recorded (Asif and Rasool, 2016 Dapper et al., 2007).

#### 2.8.3 Thrombolytic activity

Five separate Eppendorf tubes were used for each participant after blood samples (2.5 mL) were taken from 25 healthy human volunteers. After 45 min, the serum was removed from the Eppendorf tubes to provide time for thrombus development. Weighing clots in Eppendorf tubes was done. Three separate Eppendorf tubes were used for each member to apply dilutions of the aqueous-methanolic extract: 20%, 10%, and 5% (100 µL each). The fourth Eppendorf tube contained 100 µL of streptokinase (30,000 IU), whereas the fifth Eppendorf tube was used to mix distilled water—the duration for which thrombolytic activity might be sustained. The liquid was drained once the 90 min were over, and the residual clots were weighed in Eppendorf tubes again. The change from pre-to post-clot lysis was recorded as the percentage of clot lysis (Ain et al., 2018).

% clot lysis=A−B / A×100

A = clot weight before lysis.

B = clot weight after lysis.

#### 2.8.4 Platelet aggregation inhibitory activity

Blood was drawn from healthy human volunteers in sodium citrate tubes. To separate plasma with abundant platelets (PRP), the blood was spun in a centrifuge for 8 min at 800 rpm Furlanello et al., 2006. The platelet-deficient plasma (PPP) was then separated for 10 min at 3,000 rpm. The platelet concentration in PRP was adjusted to 3 × 10^8 platelets/mL. Then, a platelet aggregator agent was added to the plasma (0.3 mL), i.e., ADP (3 µM)/thrombin (0.91 U/mL) (0.03 mL). The aggregation was recorded for 5 min using a spectrophotometer at 600 nm, and then the difference in % transmission was considered the endpoint. Moreover, the test was conducted within 3 h of blood withdrawal, so they might not have become inactivated. The effect of the plant extract was expressed as a percentage inhibition (Khan et al., 2021a; Khan et al., 2022).

### 2.9 *In vivo* experiment on rats

#### 2.9.1 Anti-coagulation parameter

The aqueous-methanolic leaf extract was administered to the rats in five separate groups with varying dosages. The first three groups received 100 mg/kg, 50 mg/kg, and 25 mg/kg, respectively. The fourth group was given intravenous heparin injections at 50 units/mg concentration for 7 days, and the fifth group was treated with distilled water. The blood samples were taken from the external jugular vein of each rat group on the seventh day (dos Santos et al., 2019). PT and APTT were administered. After 7 days of therapy, we measured the BT by pricking the ear veins of each rat at 0, 30, 60, and 90-minute intervals. After each 5-second interval, a filter paper was used, allowing us to determine the impact of the treatment (De Caterina et al., 1994). Each group of rats had their CT tested by inserting capillary tubes horizontally into their marginal ear veins. At 30-second intervals, the capillary tubes were punctured until the coagulated blood thread was visible (Mizgan et al., 2022).

#### 2.9.2 Thrombolytic activity

For the thrombolytic activity, the procedure previously described by Tian et al. (2017) was adopted with slight modifications. The rats were placed in a prone position, and then their ears were disinfected with commercially available alcohol swabs. After this, 0.2–1.1 mg/kg of 40% FeCl_3_ solution was administered intravenously to the marginal veins of rats’ ears (Majumdar et al., 2016). The animals were monitored after 24 h of thrombus formation. The optimal presence of the FeCl_3_ was observed based on the wine-coloured appearance in the ear vein of rats, which indicated the formation of the thrombus. The rats in the test groups were administered 200, 300, and 600 μg/kg of the different extract concentrations. The positive control group received a 100 μg/kg dose of streptokinase. After 30 min of induction of FeCl_3_ solution, the length of the clot region was measured (Mizgan et al., 2022). Then, after 24 h, the percentage of thrombus lysis after administration of thrombolytic agents and test drug was calculated using this formula:

$$\%\text{ of thrombus}=100\;/\;\left[\text{Thrombus length }\left(\text{control rats}\right)\times \text{Thrombus length }\left(\text{treated rats}\right)\right]$$

### 2.10 Antioxidant activity

Each of the four groups of six rats consisted of twenty-four Wistar rats. A 0.9% saline (NS) solution (4 mL/kg) was administered to the first group via nasal gastric tubing for 21 days. Group 2 was given doxorubicin at 10 mg/kg intraperitoneally in a single bolus injection and 4 mL/kg NS for 21 days using gastric tubing. Group 4 received an intraperitoneal injection of doxorubicin on the first day of the 21-day treatment. In contrast, Group 3 received an oral treatment of 400 mg/kg of *J. mollissima extract*, and Group 4 received an oral treatment of 600 mg/kg.

#### 2.10.1 Redox potential

Peroxide, superoxide dismutase (SOD), 2,2-diphenylpicrylhydrazyl (DPPH), and nitric oxide (NO) assays were used to measure antioxidant activity.

##### 2.10.1.1 DPPH assay

The DPPH test has been conducted as previously stated. The ethanol-diluted sample was combined with a 30:70 aqueous-methanolic extract from *Jatropha mollissima* to attain an ultimate quantity of 5 mL with varied concentrations (4 mL). Subsequently, this mixture was kept in the dark for 40 min. The absorbance of the designated solution at 517 nm was measured using a spectrophotometer. Every experiment was repeated thrice, and the amount of inhibition in terms of ascorbic acid equivalency was quantified (Iqbal et al., 2021b; Ain et al., 2023). The equation below was used to compute the percentage of DPPH scavenging potential:

$$\%=A\;\left(\text{blank}\right)-B\;\left(\text{sample}\right)/A\;\left(\text{blank}\right)\times 100$$

##### 2.10.1.2 Quantification of NO scavenging ability

An aqueous-methanolic solution containing 10 mg/mL of J. mollissima was extracted. To reach concentrations of 1,000 and 2000 μg/mL, distilled water was used to dilute the ascorbic acid and extract. A constant temperature of 4 °C was maintained for the experimental solutions. The procedure utilises a recently synthesised Griess reagent. To determine the optimal extract concentration (1,000 and 2000 g/mL), 1 mL of each concentration was combined with 0.5 mL of sodium nitroprusside (10 mM) in phosphate-buffered saline. The mixture was then incubated at 25 °C for 3 hours. To the same volume of extract, add a freshly prepared Griess reagent. The control samples did not contain the extracts, but the amount of buffer was maintained constant. Although the different coloured tubes had sufficient extracts, sodium nitroprusside was absent. One hundred 50 L of the reaction mixture were placed into a 96-well plate. We utilised a UV-Vis microplate reader (Alibaba, Hangzhou, China) to measure the absorbance at 546 nm, as described in our previous publications (Khan et al., 2021b; Iqbal et al., 2021a; Khan et al., 2022; Iqbal et al., 2022; Ain et al., 2023). Ascorbic acid and extract % NO scavenging activity and standard % inhibition were calculated in this investigation using the following formula.

% NO scavenging activity=Blank−sample/blank×100

##### 2.10.1.3 Peroxide radical scavenging assay

Our tests were carried out by modifying an existing approach, which involved preparing extract and standard solutions at two distinct concentrations (1,000 and 2000 μg/mL), along with a 43 mM H_2_O_2_ solution in a 0.1 M phosphate buffer (pH 7.4). Next, add the H_2_O_2_ solution (43 mM) at a volume of 0.6 mL after mixing the sample solutions with the 3.4 mL of phosphate buffer. The absorbance at 230 nm was measured using a UV spectrophotometer. A sodium phosphate buffer, not mixed with hydrogen peroxide, was used as a control (Ain et al., 2023). The formula employed for calculating scavenging efficiency in % H_2_O_2_ is as follows:

% Inhibition:blank−sample / blank×100

##### 2.10.1.4 Reducing power scavenging assay

Many people utilize phenolic antioxidant activity as a surrogate for their ability to decrease iron (III). A technique similar to that suggested by Khan et al. (2021a) and Iqbal et al. (2021b) was used to determine the reducing potential of the extracts. After the extracts were diluted in water to a concentration ranging from 1,000 to 2000 μg/mL, they were mixed with 2.5 mL of 1% potassium ferricyanide and 2.5 mL of 0.2 M phosphate buffer at pH 6.6. All the ingredients were boiled for 20 min at 50 °C. The procedure was halted after adding 2.5 mL of 10% trichloroacetic acid and centrifuging at 3,000 rpm for 10 min. To quantify the absorbance at 700 nm, 2.5 mL of the top solution layer was combined with 2.5 mL of distilled water and 0.5 mL of FeCl3 (0.1%). An increase in the absorbance of the reaction mixture indicated a rise in the reducing power. Ascorbic acid served as an effective surrogate for positive control (Ain et al., 2023).

##### 2.10.1.5 Superoxide dismutase assay

Assessed the herb’s capacity to scavenge superoxide anion radicals employing a modified methodology based on the findings of Iqbal et al. (2022). The PMS, NADH, and NBT framework generated superoxide radicals. In a test tube containing 625 µL of Tris-HCl buffer (16 mM, pH 8.0), incorporate 50 µL of the test substances at different dilutions, together with 125 µL of NBT (300 mM) and 125 µL of NADH (468 mM). Once the mixture had been introduced to 125 µL of PMS (60 mM), the reaction began. The mixture is vigorously mixed before incubating at room temperature for 5 minutes (Khan et al., 2021b). Spectrophotometer readings were taken using a Hitachi U-1900 UV/VIS instrument from Hitachi High-Technologies Corporation to analyse the absorbance.

#### 2.10.2 Assessment of cardiac biomarkers

The CK-MB, LDH, Troponin I, serum sodium, and potassium levels were measured by taking blood samples using the retro-orbital method on the zero and 21st days. The animals were weighed and anaesthetized with a combination of xylazine and ketamine (1:10) on day 21 following a 12-hour fast. The blood was drawn and clotted for testing using a heart puncture. The serum was separated by centrifugation for 15 min at 3,000 rpm as previously reported method (Manzoor et al., 2022; Farooq et al., 2023).

#### 2.10.3 Assessment of cardiotoxicity by biochemical serum parameters

Standard enzymatic kits that are commercially available were used to quantify the serum concentrations of creatinine kinase myocardial band (CK-MB), lactate dehydrogenase (LDH), and troponin-I. We also checked the levels of electrolytes in the blood, such as potassium and sodium.

##### 2.10.3.1 Computation of CK-MB

CK-MB is a highly sensitive biomarker for the detection of myocardial infarction and toxic drug effects. Its blood levels can be measured using CK-MB assay kits. Animals treated with doxorubicin were evaluated for their CK-MB levels using an SBio test kit. Following receipt of blood samples on or after the 21st day, the following protocol was followed: incubation for 20 min, centrifugation to extract serum, and analysis at 340 nm with a UV spectrophotometer. For the experiment, 1 mL of working reagent had to be heated to 37 °C for 1 min before 0.05 mL of the sample could be added. Once the mixture has been blended for 10 min, measure the initial absorbance (A0) and repeat the process at 1, 2, and 3-minute intervals. (Khan et al., 2022; Iqbal et al., 2022). In order to determine the mean rate of variation in absorbance (ΔA/min), please apply the given formula.

$$\text{CK}-\text{MB activity in }U/L\;37^{0}C=\Delta A/\min.\times 6666$$

##### 2.10.3.2 Estimation of serum LDH

A human kit assessed lactate dehydrogenase (LDH) by collecting blood samples at 0, 10, and 21 days and letting them clot for 20 min. After that, the clotted blood was spun at 3,000 rpm for 15 min in a centrifuge. After separating the blood from the serum, it was collected. A UV spectrophotometer operating at 340 nm was used to examine the serum following the following protocol (Iqbal et al., 2022). The absorbance readings are obtained by calculating each minute’s average absorbance variation, denoted as ΔA/min. The LDH activity can be measured by multiplying the sample’s ΔA/min by 8,095.

$$U/l\;\left(25^{0},30^{0}\right)=\Delta \;A/\min.\;X$$

Where X is the LDH value, and is measured in triplicate.

##### 2.10.3.3 Assessment of serum sodium and potassium

Centrifuging the blood at 3,000 rpm for 15 min separated the serum, allowing us to determine sodium and potassium concentrations. A flame photometer (Sherwood Model 410, United Kingdomc) was used to evaluate the sample after it had been diluted 1:200. Its authenticity was guaranteed by repeating the entire process twice (Iqbal et al., 2022).

#### 2.10.4 Vasodilator activity

The Aorta from all the group was surgically anatomized from the thoracic cavity. Then, aortic slices (length: 3–4 mm) were immersed in an organ bath (10 mL) containing the Krebs-Henseleit solution, then continually incubated with 95% carbogen gas (O_2_ 95% and CO_2_ 5%). The bottom knob was fixed, and the higher knob to a pressure-displacement sensor that was linked to Power lab data assortment device to capture the isometric deflations. To prevent metabolite accumulation, Kreb solution was reloaded every 15 min during the 40-minute steadiness phase at 1 g inactive strain (Khan et al., 2014). The aorta tissue preparation was pre-constructed with 1 × 10^−6^ M phenylephrine (PE) after equilibration till stable contractile curvature (5–8 min) was attained, and the vasodilatory effect of *J. mollissima* was assessed using a collective dosing approach (Khan et al., 2016).

#### 2.10.5 Calcium channel blocking activity

*J. mollissima* was compared to persistent deflations induced by K+ (conc. 80 mM) in the secluded aorta due to activation of the Ca^++^ channels (voltage-dependent), which initiated contractile feedback via extracellular Ca^++^ invasion. Chemicals that impede the inflow of Ca++ ions through these channels can diminish K-80-induced deflations. An additive test material administration is necessary for a concentration-dependent inhibitory response against prolonged contractions (Saqib et al., 2022; Khan et al., 2022). The relaxant potential of the test medication on induced deflations was stated as a percent response of the deflation of the control group (Gilani et al., 1994).

### 2.11 Effect of *J. mollissima* on body weights/heart weight

Animal weight was documented at 0 and 21st. The rats were given intraperitoneal injections of doxorubicin at a dose of 10 mg/kg on the first day of the 21-day experiment. Then, the heart-to-body weight ratio was measured for the identification of cardiac weight index and necrosis (Khan et al., 2021a).

### 2.12 Real-time polymerase chain reaction (RT-PCR)

Total RNA was extracted using a commercial RNA kit (Vazyme Biotech Co., Ltd., Beijing, China). A UV spectrophotometer measured RNA integrity and concentration. Purified RNA (2 μg) was used for reverse transcription to synthesize cDNA utilizing a high-efficiency reverse transcription kit. The synthesized cDNA served as a template for PCR amplification using SYBR Green (Tiangen Biotech Co., Ltd., Beijing, China). Gene-specific primers were employed targeting TNF-α, IL-1β, IL-6, IL-10, and 18S rRNA. Relative mRNA expression was calculated using the 2^−ΔΔCt^ method. Each reaction was performed thrice to ensure reproducibility.

### 2.13 Statistical evaluation

After being analyzed using one-way ANOVA, the results were presented as the mean ± SEM, followed by Dunnett’s multiple comparison tests (for anticoagulant and thrombolytic activities), and Bonferroni *post hoc* test (for antioxidant activities). A confidence interval of 95% was calculated using SPSS, and a p-value less than 0.05 was deemed significant.

## 3 Results

### 3.1 Preliminary phytochemical screening

The results of the phytochemical analysis of the *J. mollissima* aqueous-methanolic leaves extract are shown in Table 1.

**TABLE 1:** Phytochemical screening of aqueous-methanolic leaf extract of *J. mollissima*

Phytochemicals	Results
Alkaloids	+
Anthracenic derivatives	++++
Anthocyanins	+++
Anthraquinones	+++
Phenolic metabolitess	+++
Coumarins	+++
Saponins	++
Tannins	+++
Triterpenes/sterols	++
Flavonoids	++++

+ = present and ++ = highly present.

### 3.2 HPLC analysis

**FIGURE 1:**
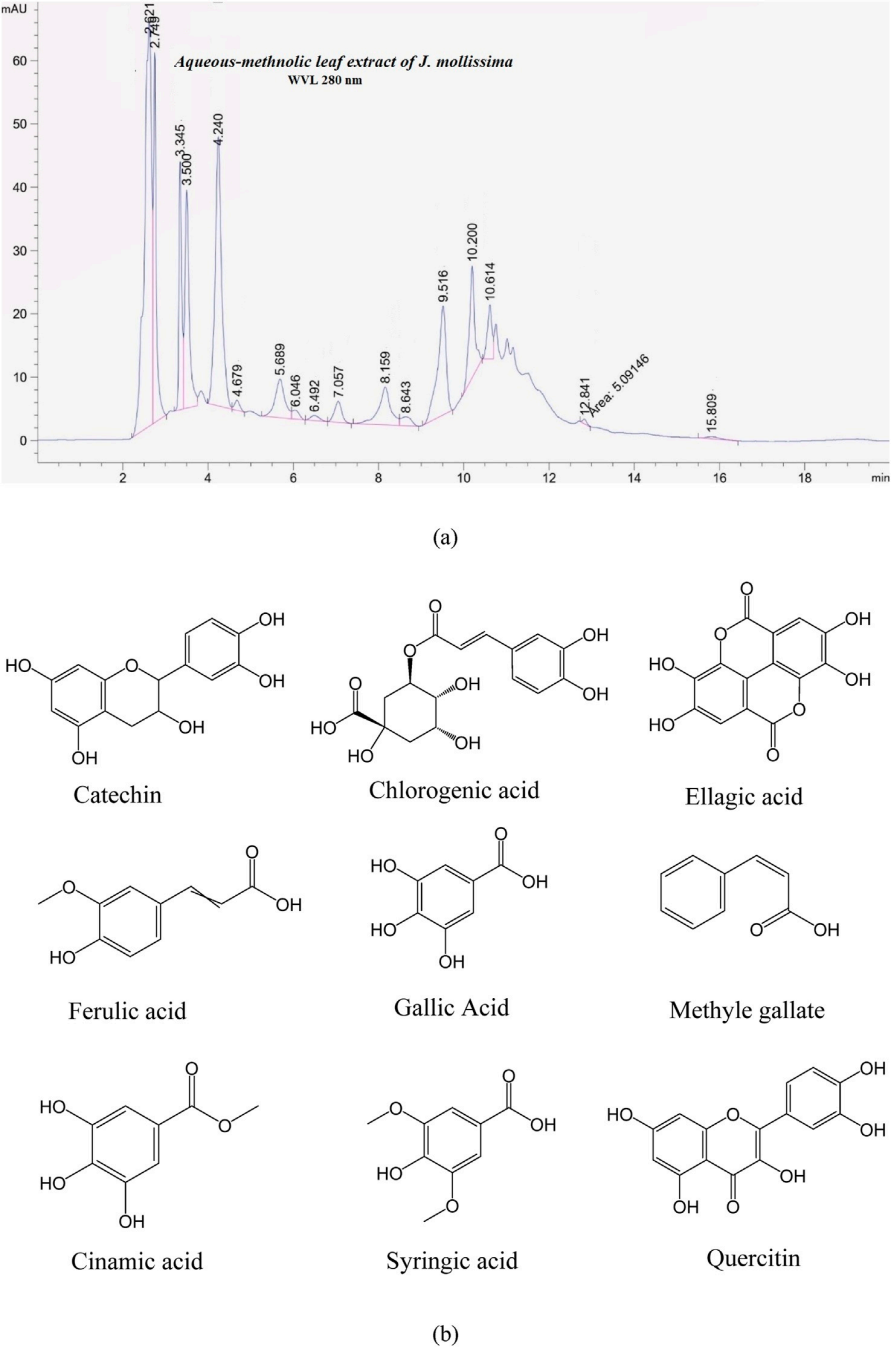
**(a)** Shows an HPLC of aqueous-methanolic leaves extract of *J. mollissima,* while **(b)** shows the structure found in aqueous-methanolic leaves extract of *Jatropha mollissima*

The HPLC results of the *J. mollissima* aqueous-methanolic leaves extract are shown in Figure 1.

### 3.3 Impact on *in vitro* activities

#### 3.3.1 Antithrombotic effect

**FIGURE 2:**
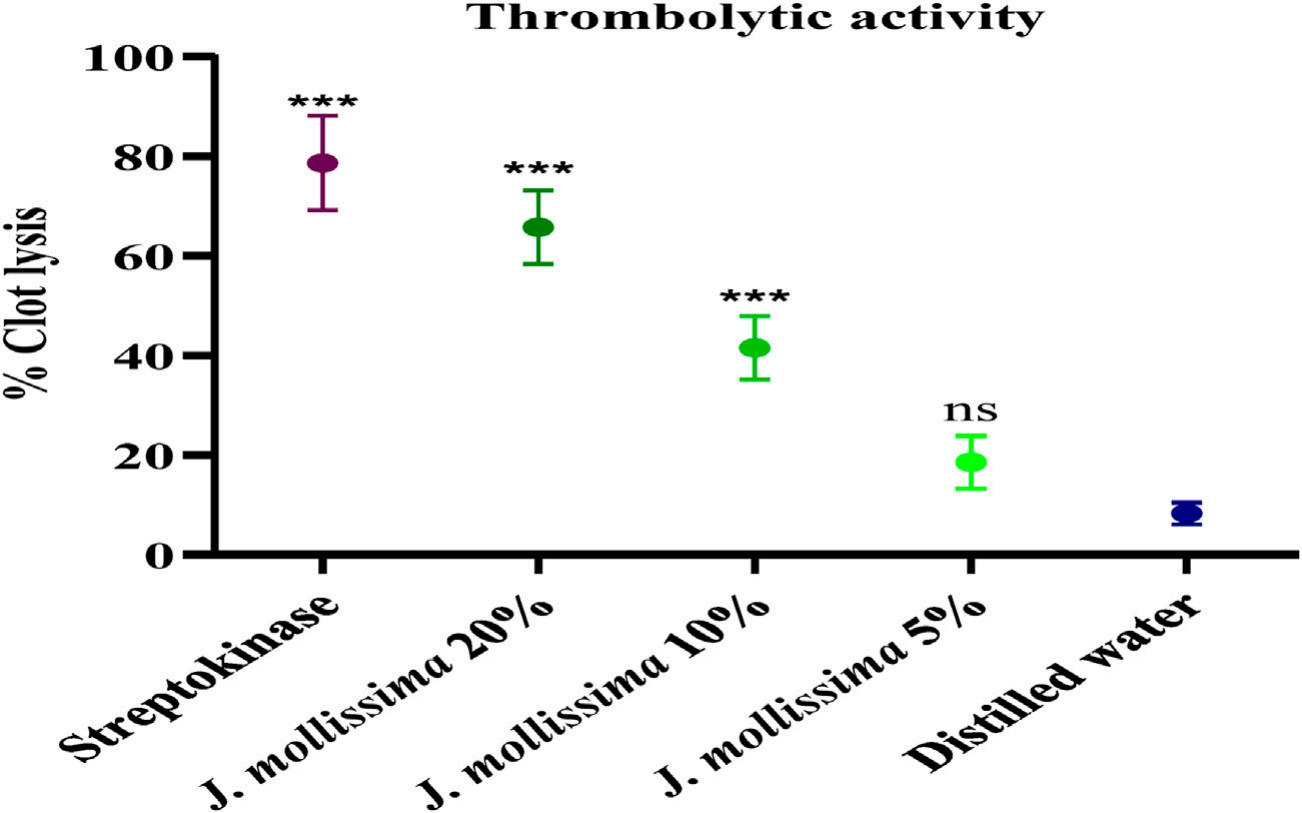
Antithrombotic effect of aqueous-methanolic leaves extract of *J. mollissima.* One-way ANOVA was performed for the statistical analysis, comparisons among different groups was carried out by Dunnett’s multiple comparison test; **p < 0.005 and ***p < 0.0001 (n = 5).

The clot lysis was substantial (p < 0.01–0.001) in the 20% and 10% diluted aqueous-methanolic *J. mollissima* leaves extracts. Still, it was found insignificant (p < 0.1) in the 5% diluted extract, as shown in Figure 2.

#### 3.3.2 Anticoagulant activity

**FIGURE 3:**
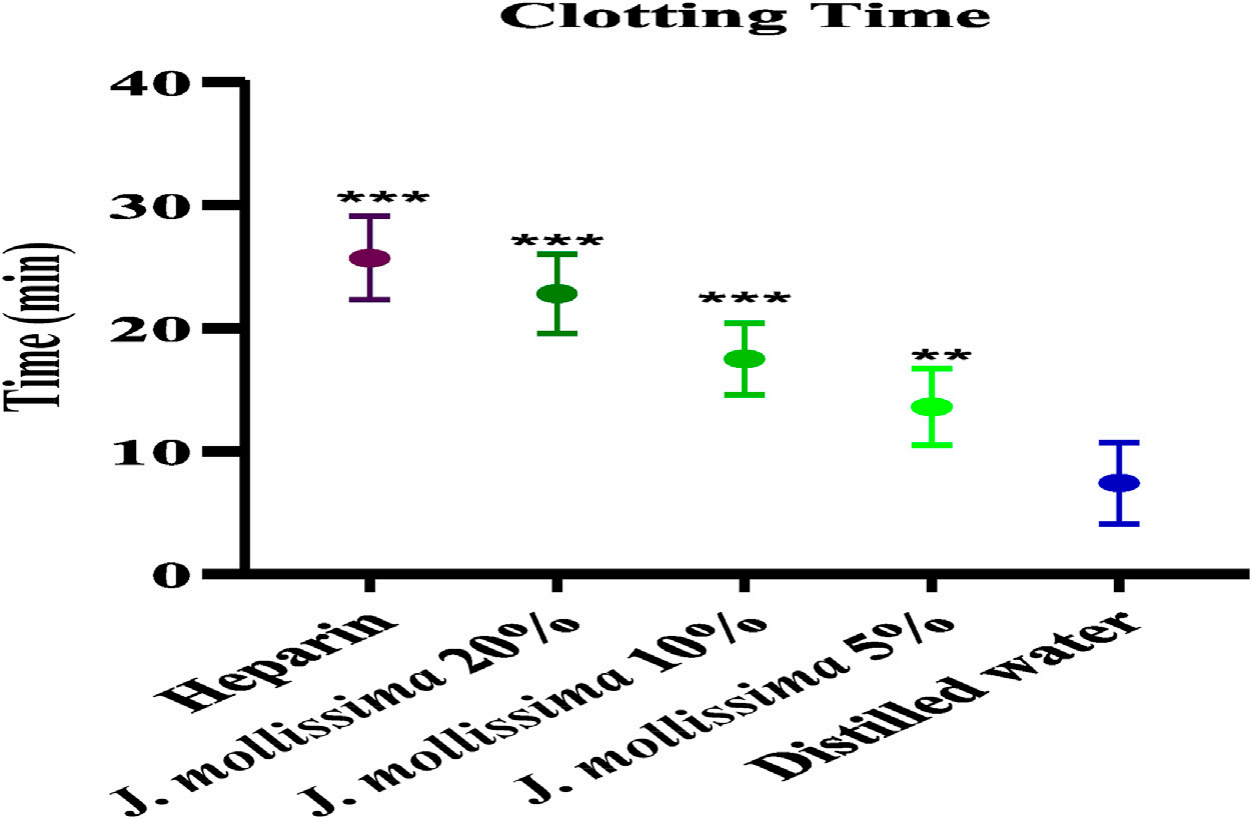
Anticoagulant activity (clotting time). One-way ANOVA was performed for the statistical analysis, comparisons among different groups was carried out by Dunnett’s multiple comparison test; **p < 0.005 and ***p < 0.0001 (n = 5).

Figure 3 displays the considerable increase (p < 0.001) in CT at 20%, 10%, and 5% dilutions of the aqueous-methanolic *J. mollissima* leaves extract.

#### 3.3.3 Prothrombin time and activated partial thromboplastin time

**FIGURE 4:**
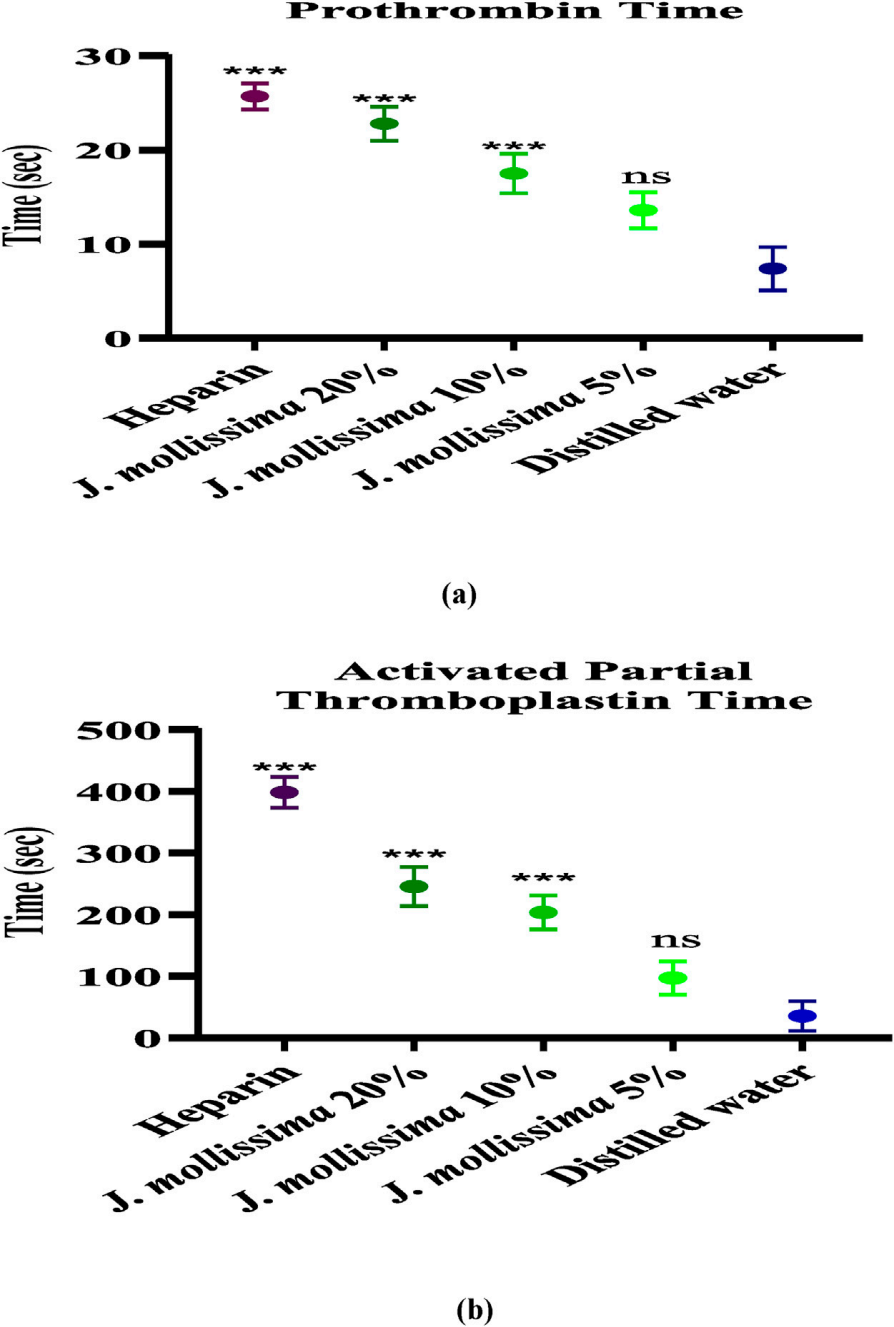
**(a)** Prothrombin time; **(b)** activated partial thromboplastin time. One-way ANOVA was performed for the statistical analysis, comparisons among different groups was carried out by Dunnett’s multiple comparison test; **p < 0.005 and ***p < 0.0001 (n = 5).

The Prothrombin time and activated partial thromboplastin time results given in Figure 4 indicate that the aqueous-methanolic seed extract of *J. mollissima* exhibits a considerable (p < 0.01–0.001) increase at 20% and 10% dilutions but an insignificant (p < 0.1) increase at 5% dilution.

### 3.4 Effect on several coagulation markers following 1-week administration in rats

**FIGURE 5:**
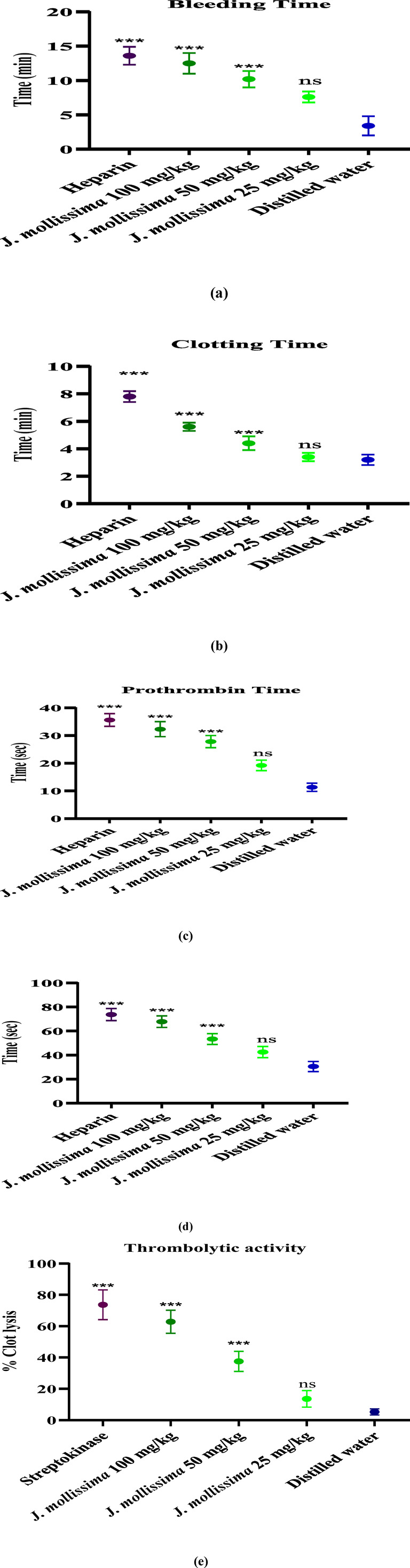
Effect on several coagulation markers following 1-week administration in rats. **(a)** Bleeding time; **(b)** Clotting time; **(c)** Prothrombin time; **(d)** activated partial thromboplastin time; **(e)** Thrombolytic activity. One-way ANOVA was performed for the statistical analysis, comparisons among different groups was carried out by Dunnett’s multiple comparison test; **p < 0.005 and ***p < 0.0001 (n = 5).

The aqueous-methanolic leaves extract of *J. mollissima* demonstrated a significant increase (p < 0.01–0.001) in BT, CT, APTT, and PT at concentrations of 100 mg/kg and 50 mg/kg while showing a negligible increase (p < 0.1) at a dose of 25 mg/kg, as illustrated in Figure 5.

### 3.5 Platelet aggregation inhibitory effect

**FIGURE 6:**
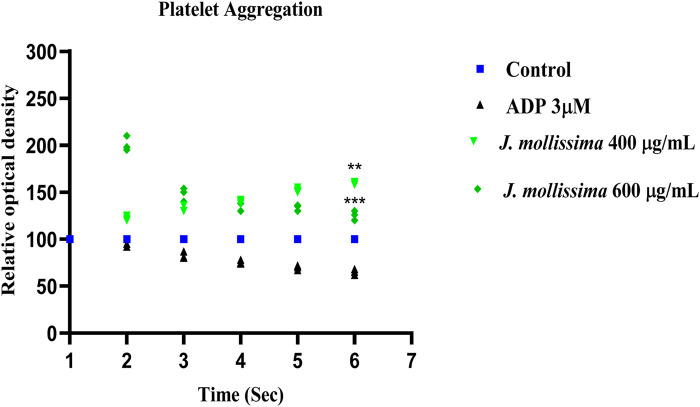
Dose dependent platelet aggregation inhibitory effect of *J. mollissima.* One-way ANOVA was performed for the statistical analysis, comparisons among different groups was carried out by Dunnett’s multiple comparison test; **p < 0.005 and ***p < 0.0001 (n = 5).

The Aqueous-methanolic extract of *J. mollissima* showed significant platelet aggregation inhibitory effect dose-dependently induced by ADP, as shown in Figure 6.

### 3.6 Antioxidant assays

Data from antioxidant assays reveal that *J. mollissima* aqueous-methanolic leaves extract significantly reduced DPPH, reducing power, NO, H_2_O_2_, and SOD levels, with the effect being dosage-dependent (Table 2).

**TABLE 2:** Findings from various antioxidant assays conducted on *J. mollissima* plant extract.

Concentration (μg/mL)	Extract inhibition					Standard inhibition				
	DPPH	Reducing power	NO	H_2_O_2_	SOD	DPPH	Reducing power	NO	H_2_O_2_	SOD
**1,000**	85.70	71.20	90.11	86.85	78.39	95.24	79.26	90.83	93.00	93.25
**2000**	92.50*	84.02*	92.05*	89.78*	83.63*	94.14	85.15	86.32	90.25	91.26

The results were reported as mean ± SEM, and analyzed using one-way ANOVA, followed by Bonferroni *post hoc* test. * shows p value less than 0.01 when compared to positive control.

### 3.7 Cardioprotective effect of *Jatropha mollissima* against doxorubicin-induced toxicity

#### 3.7.1 Effect of *J. mollissima* on body weights of animals

Day 0 body weight did not differ significantly between these groups (p > 0.05). Rats given doxorubicin experienced weight loss for the course of the trial, in comparison to their weight on the first day (p < 0.05). All groups treated with *J. mollissima*, especially those given 400 and 600 mg/kg, showed weight gains (p < 0.05).

**FIGURE 7:**
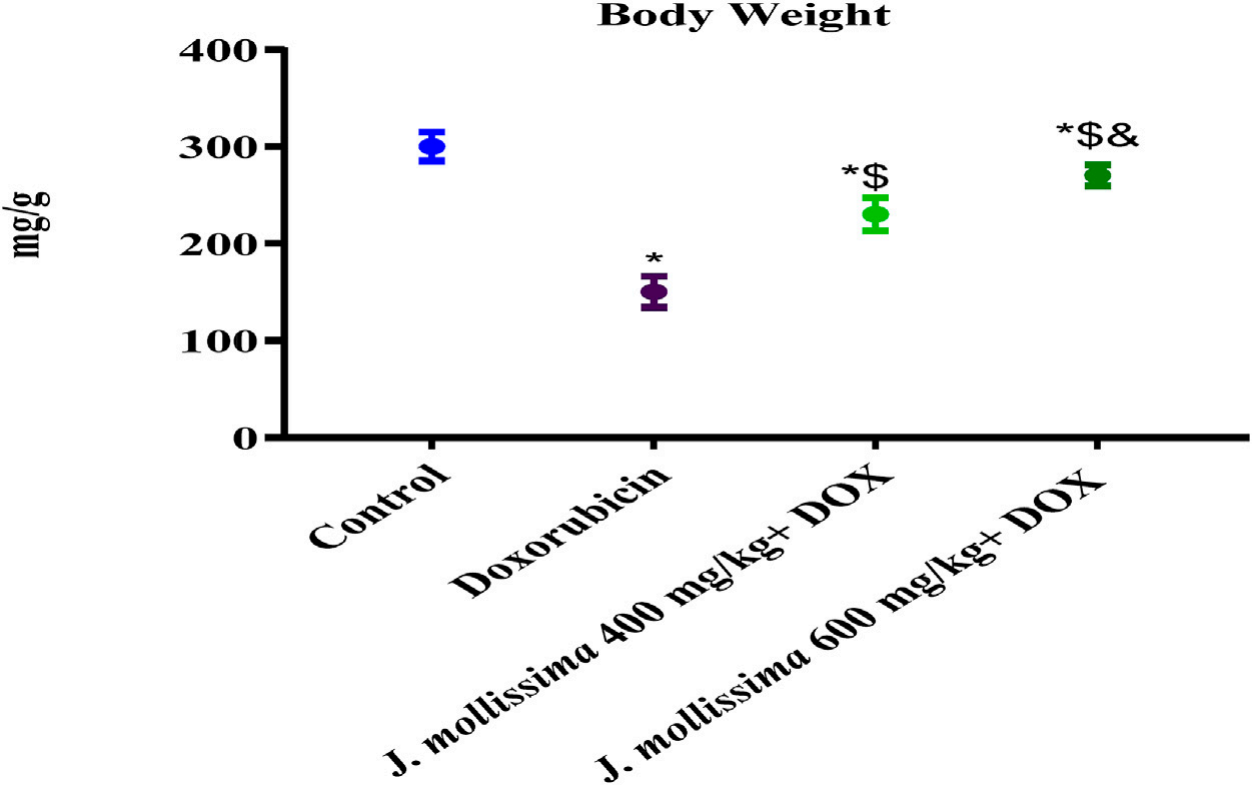
B.W of control, doxorubicin, doxorubicin + *J. mollissima* 400, and doxorubicin + *J. mollissima* 600 mg/kg. Results are deemed to be statistically significant (*) if *p* is less than 0.005. For each day, a * denotes statistical significance when compared to the control group, $ to Doxorubicin, and & to Dox + *J. mollissima* 400.

Finally, a weight gain was observed in both the control and *J. mollissima* rat groups. In contrast, rats given Dox had a pattern of decreasing body weight, likely due to reduced hunger and reduced food consumption. On the other hand, animals that were exposed to *J. mollissima* intoxicated with doxorubicin showed a substantial rise in body weight, as shown in Figure 7.

#### 3.7.2 Heart weight

In contrast to the control group, rats treated with doxorubicin had significantly lower heart weights. As demonstrated in Table 3, a slight increase in heart weight was observed when different doses of *J. mollissima* extracts were treated with doxorubicin, namely, 400 and 600 mg/kg, respectively.

**TABLE 3:** The impact of *J. mollissima* on changes in heart weight caused by doxorubicin.

Groups	Heart weight (g)
Control	0.89 ± 0.49
DOX	0.59 ± 0.48 (*)
Doxorubicin + *J. mollissima* 400 mg/kg	0.65 ± 0.49 (#)
Doxorubicin + *J. mollissima* 600 mg/kg	0.69 ± 0.46 ($)

Results are deemed to be statistically significant (*) if p is less than 0.005. For each day, a * denotes statistical significance when compared to the control group, $ to Doxorubicin, and # to Dox + *J. mollissima* 400.

### 3.8 Effect of *J. mollissima* on cardiac marker enzymes

#### 3.8.1 *J. mollissima* impact on (CK-MB)

**FIGURE 8:**
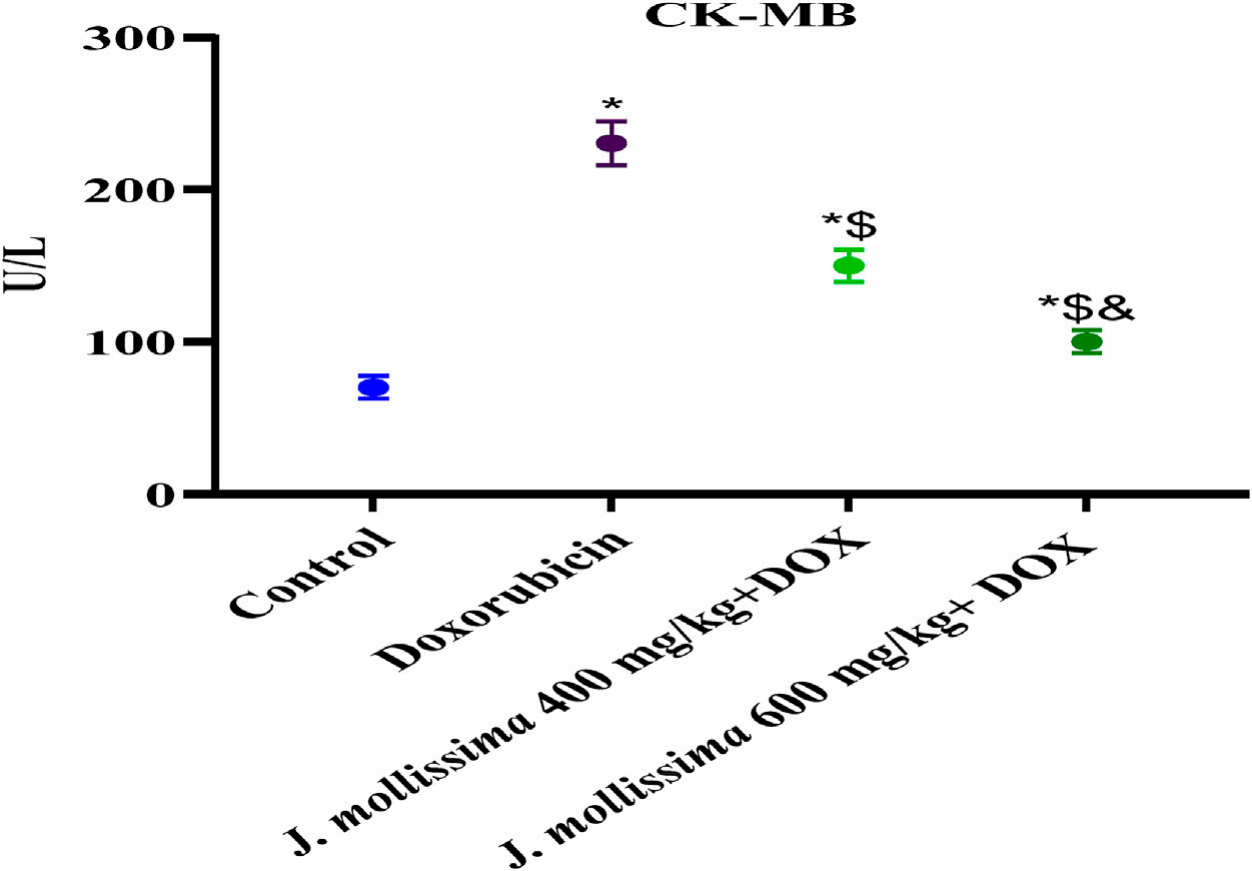
Blood CK-MB levels of healthy controls, doxorubicin, and doxorubicin + *J. mollissima* at 400 and 600 mg/mL, respectively. Results are deemed to be statistically significant (*) if *p* is less than 0.005. For each day, a * denotes statistical significance when compared to the control group, $ to Doxorubicin, and & to Dox + *Jm* 400.

The rats who were given doxorubicin had significantly higher serum levels of CK-MB (p > 0.05) in comparison to the control group (p < 0.05). Figure 8 shows that the concentrations of CK-MB gradually decreased (p < 0.05) when *J. mollissima* was administered to the 400 mg/kg and 600 mg/kg treatment groups.

#### 3.8.2 Effect of *J. mollissima* on serum lactate dehydrogenase (LDH)

**FIGURE 9:**
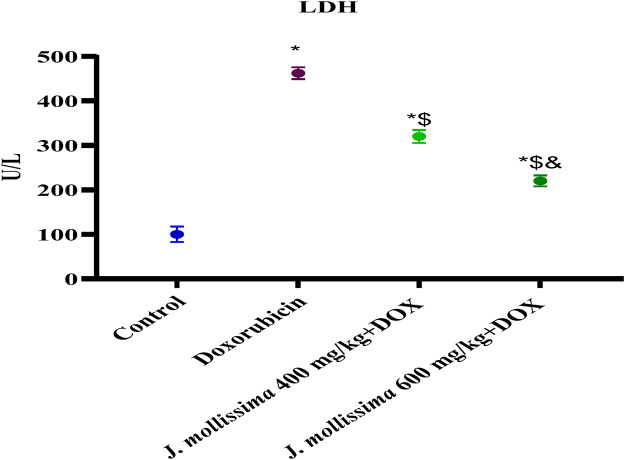
Blood LDH levels of healthy controls, doxorubicin, and doxorubicin + *J. mollissima* at 400 and 600 mg/mL, respectively. Results are deemed to be statistically significant (*) if *p* is less than 0.005. For each day, a * denotes statistical significance when compared to the control group, $ to Doxorubicin, and and to Dox + *Jm* 400.

The rats who were given doxorubicin had significantly higher serum levels of LDH (p > 0.05) compared to the control group (p < 0.05). Figure 9 shows that the concentrations of LDH were markedly reduced (p < 0.05) in the treatment groups receiving 400 and 600 mg/kg after being administered *J. mollissima.*

#### 3.8.3 Effect of *J. mollissima* on troponin I (TnI)

**FIGURE 10:**
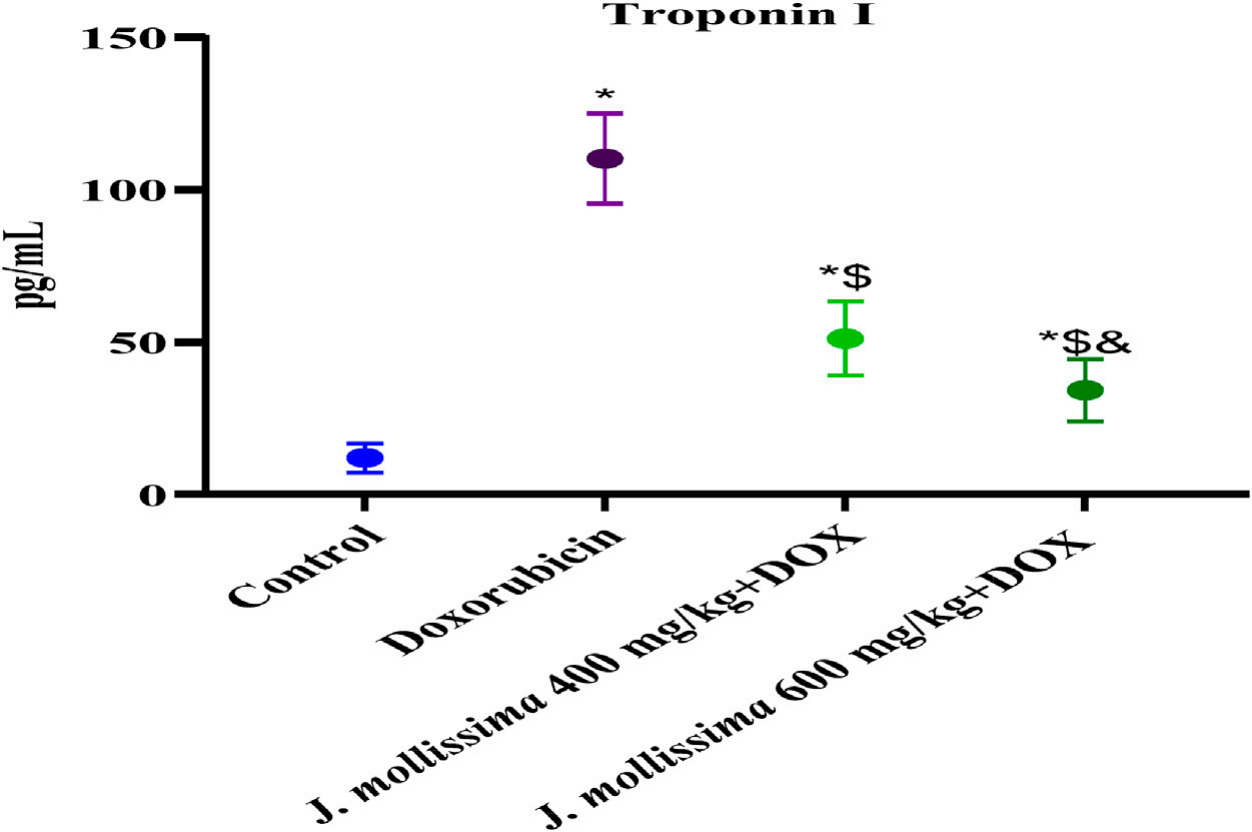
Serum Troponin I (TnI) levels of control, doxorubicin, doxorubicin + *J. mollissima* 400, and doxorubicin + *J. mollissima* 600. Results are deemed to be statistically significant (*) if *p* is less than 0.005. For each day, a * denotes statistical significance when compared to the control group, $ to Doxorubicin, and & to Dox + *Jm* 400.

Troponin I levels were significantly higher in rats that were treated with doxorubicin (p > 0.05) when contrasted with the control group (p < 0.05). Figure 10 shows that after *J. mollissima* was administered, Troponin I (TnI) levels in both the 400 and 600 mg/kg treatment groups decreased significantly (p < 0.05).

#### 3.8.4 The effect of *J. mollissima* on blood sodium levels

**FIGURE 11:**
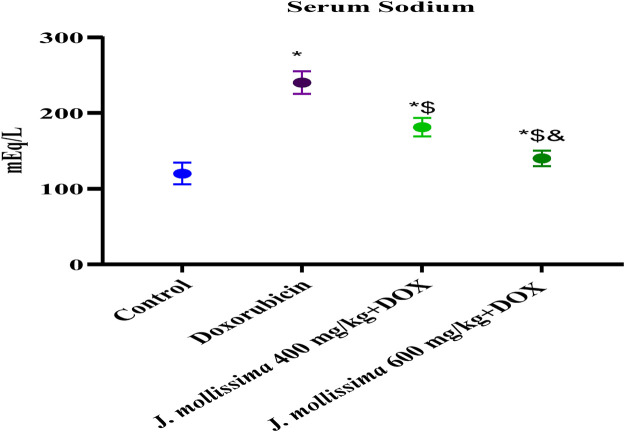
Concentrations of sodium in the blood of healthy controls, doxorubicin, and doxorubicin + *J. mollissima* at 400 and 600 mg/mL, respectively. Results are deemed to be statistically significant (*) if *p* is less than 0.005. For each day, a * denotes statistical significance when compared to the control group, $ to Doxorubicin, and & to Dox + *Jm* 400.

After administering 10 mg/kg of doxorubicin on day one, the control group’s serum sodium levels were lower than those of the group administered doxorubicin. Sodium levels dropped significantly (p < 0.05) in the groups administered 400 and 600 mg kg of *J. mollissima,* as shown in Figure 11.

#### 3.8.5 The effect of *J. mollissima* on blood potassium levels

**FIGURE 12:**
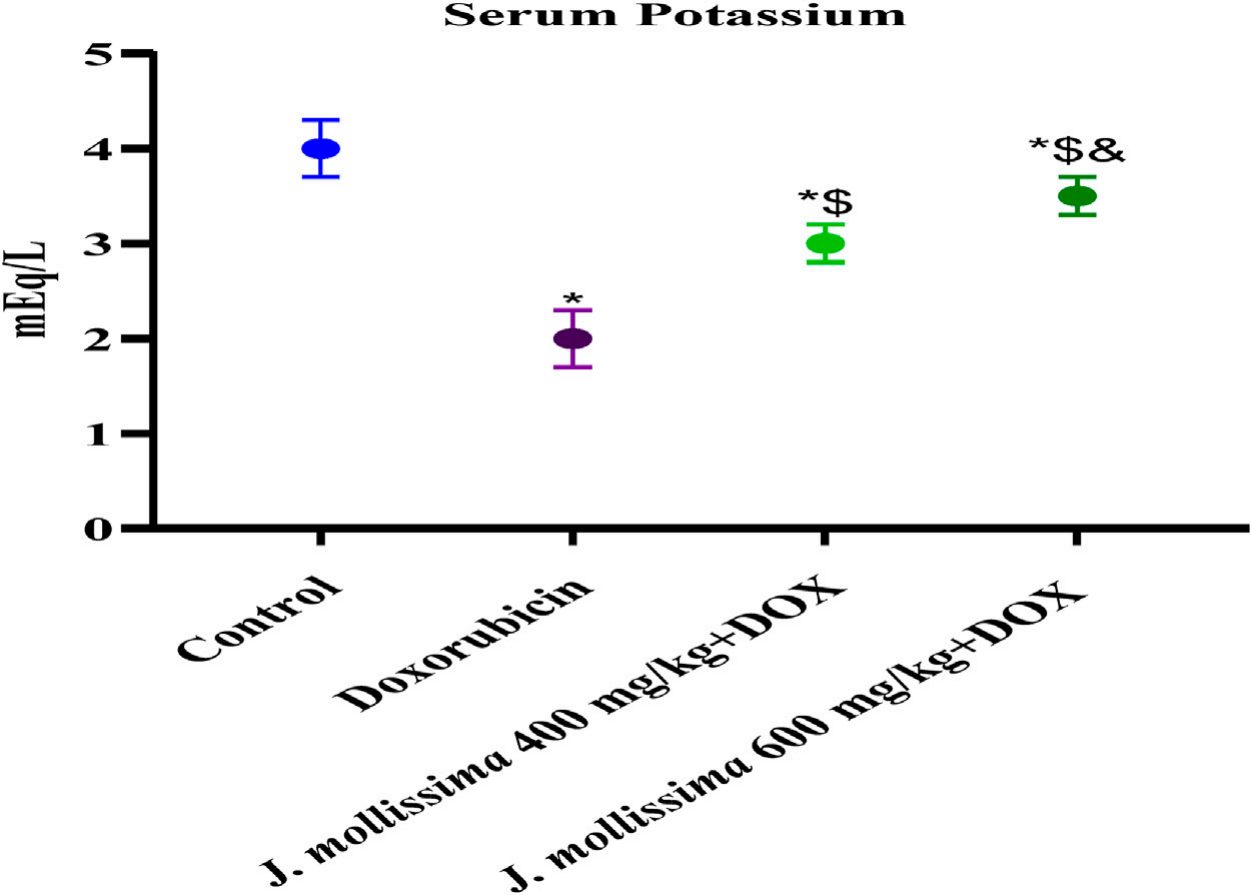
Concentrations of potassium in the blood of healthy controls, doxorubicin, and doxorubicin + *J. mollissima* at 400 and 600 mg/mL, respectively. Results are deemed to be statistically significant (*) if *p* is less than 0.005. For each day, a * denotes statistical significance when compared to the control group, $ to Doxorubicin, and and to Dox + *Jm* 400.

The cohort administered a single dose of 10 mg/kg of doxorubicin on the first day exhibited lower serum potassium levels than the control group (p > 0.05). Figure 12 demonstrates that the groups given 400 and 600 mg kg of *J. mollissima* had somewhat higher sodium levels (p < 0.05).

#### 3.8.6 Vasodialatory effect of *J. mollissima* leaves extract

**FIGURE 13:**
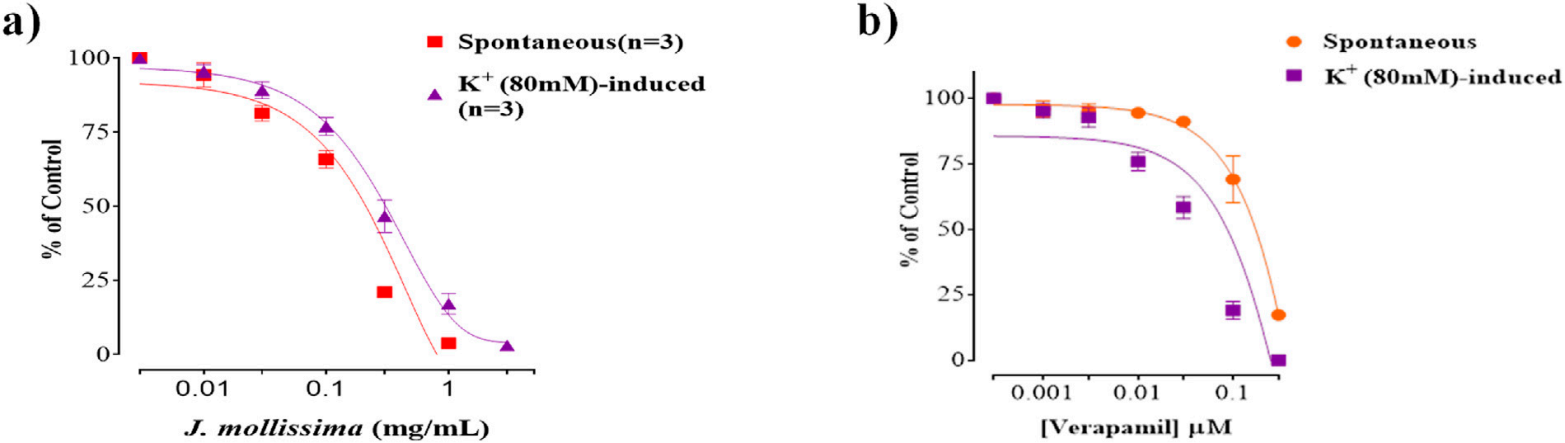
Vasodilatory effect of aqueous methanolic leaf extract of *J. mollissima* against spontaneous and K 80 induced contraction **(a)**, and comparative vasodilatory effect of verapamil **(b)**. All the values (n = 5) are depicted as mean ± SEM.

Aquous methanolic leaf extract of *J. mollissima* showed dose dependent relaxation of spontaneous and K 80 induced vasoconstriction in islated sortic strip of rabbit*.* Similar vasolaxant effect was shown by the standard vasorelaxant drug verapamil in islolated aortic strip (Figure 13).

#### 3.8.7 Calcium channel blocking effect of *J. mollissima*

**FIGURE 14:**
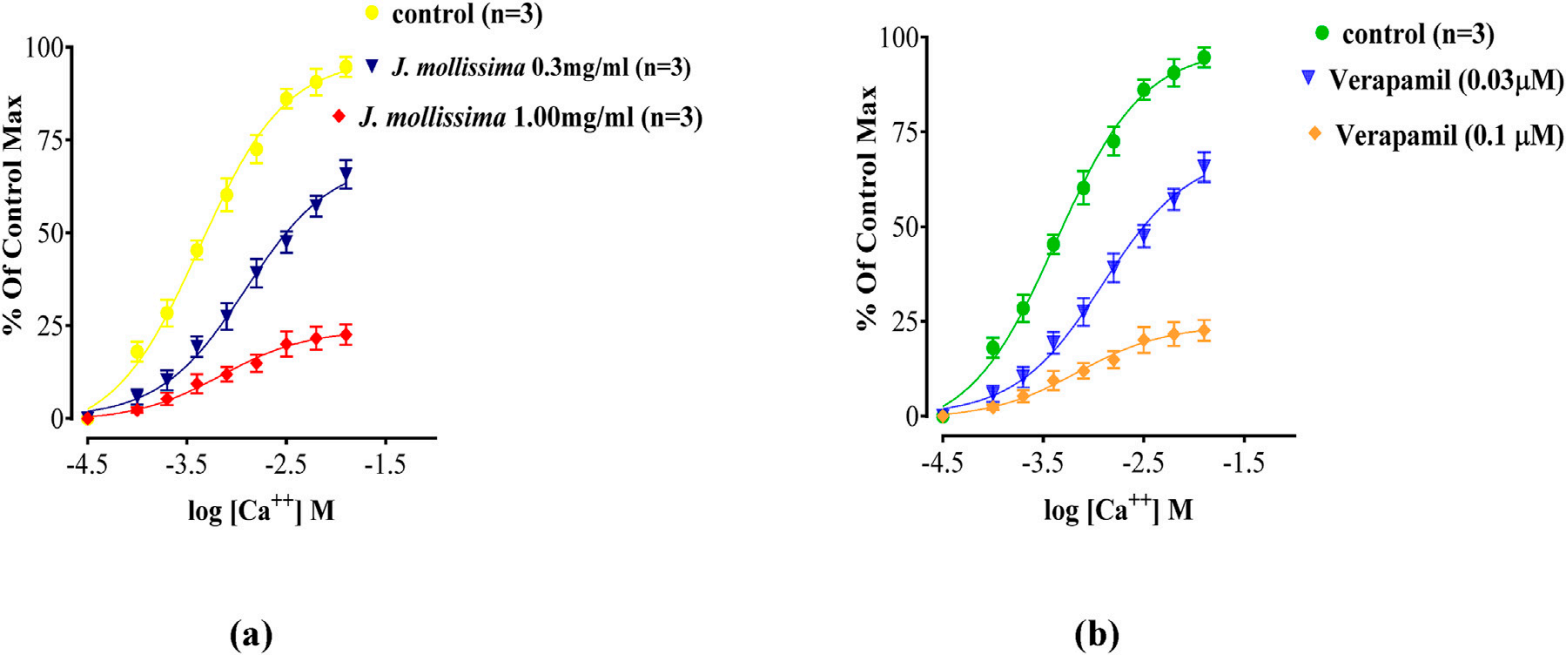
Calcium channel blocking effect of aqueous methanolic leaf extract of *J. mollissima* **(a)**, and comparative calcium channel blocking effect of verapamil **(b)**. All the values (n = 5) are depicted as mean ± SEM.

Aquous methanolic leaf extract of *J. mollissima* showed dose dependent voltage gated calcium channel blocking effect in islated sortic strip of rabbit*.* Similar voltage gated calcium channel blocking effect was shown by the standard calcium channel blocker drug verapamil in islolated aortic strip (Figure 14).

### 3.9 Jm Regulates cardiac inflammatory markers in doxorubicin rats

**FIGURE 15:**
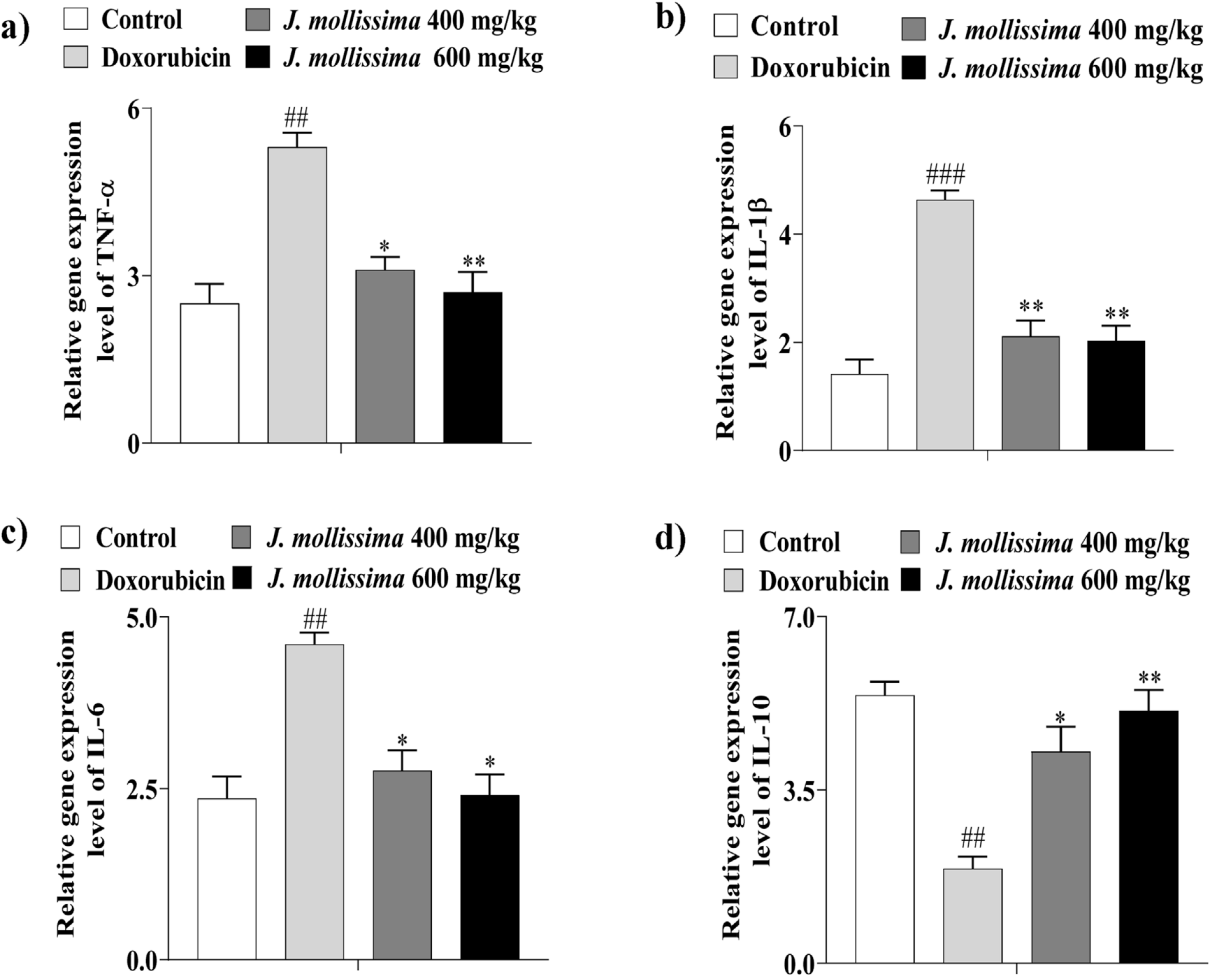
Effects of Jm aq. Extract on proinflammatory and anti-inflammatory cytokines. **(a)** Changes in the levels of TNF α. **(b)** Changes in the levels of IL-1β. **(c)** Changes in the levels of IL-6. **(d)** Changes in the levels of IL-10. The data are presented as mean ± SEM. Comparisons among the doxorubicin group and control are represented through ^##^ *p* < 0.01 and ^###^ *p* < 0.001. Treatments versus doxorubicin contrasts are specified by ^*^, ^**^, consistent to *p* < 0.05, *p* < 0.01 respectively. Each experimental group comprised four rats.

To assess whether the *J. mollissima* aq. extract possesses anti-inflammatory properties, we quantified the brain levels of TNF-α, IL-1β, IL-6, and the anti-inflammatory cytokine IL-10 in cardiotoxic rats. TNF-α expression was significantly elevated in the Doxorubicin group compared to the normal control, reflecting an intensified inflammatory response (Figure 15a). Administration of Jm extract at 400 and 600 mg/kg effectively reduced TNF-α levels, suggesting a notable anti-inflammatory effect. A similar pattern was observed for IL-1β, which was markedly increased in cardiotoxic rats but significantly decreased following treatment with Jm at different doses (Figure 15b). IL-6 levels were also considerably higher in the doxorubicin group and were brought down significantly by both interventions (Figure 15c). In contrast, IL-10 levels were substantially lower in cardiotoxic animals; however, treatment with Jm extract led to a significant upregulation of this protective cytokine (Figure 15d). These outcomes indicate that the Jm aqueous extract helps ameliorate Doxorubicin-induced cardiac inflammation, acting through the suppression of proinflammatory cytokines and the enhancement of anti-inflammatory responses, in a manner comparable to other groups.

## 4 Discussion

Natural therapies are becoming increasingly prevalent worldwide because of their significant role in the treatment of many heart disorders and their low or nonexistent side effects. Due to its ability to scavenge free radicals, J. mollissima shows promise as an antioxidant and is widely recognized as a traditional herbal medicine and a dietary spice. Suppression of free radicals, including superoxide and hydroxyl, by *J. mollissima* extract is dose-dependent. Lipid peroxidation and protein oxidation inhibition are dose-dependent (Abogmaza et al., 2020). The body’s antioxidant enzyme levels, which include peroxidases, catalase, and superoxide dismutase, can be restored to normal with their help. This investigation aimed to ascertain whether *J. mollissima* extract protected the heart from doxorubicin-induced cardiotoxicity.

The doxorubicin-intoxicated group had a mortality rate of 40% before the experiment ended because of the severe heart toxicity. As indicated, ascites developed due to salt retention and tubular dysfunction, resulting in increased extracellular volume and fluid leakage into the interstitium (Herman et al., 1988; Olson and Mushlin, 1990; Ydegaard et al., 2019). The high death rate was presumably caused by nephrotoxicity, which was indicated by multiple renal signs. Many patients noticed they were eating less and losing weight as a result of the drug’s harmful effects on the gut mucosa. The medications accomplished this by acting indirectly on the digestive tract, which reduced appetite by decreasing the secretion of naturally occurring hormones (Zima et al., 2001). According to prior research, compared to the intoxicated group, those who used *J. mollissima* improved food consumption, both of which contributed to their weight gain (El-Far et al., 2016).

Doxorubicin created reactive oxygen species (ROS), which led to cardiac cell death by damaging lipids, DNA, and proteins and impairing cellular function. The most dependable markers of injury to cardiac cells are troponin I, lactate dehydrogenase, and the creatine isoenzyme CK-MB. Their significantly higher concentration in the Doxorubicin group suggested extensive damage to heart cells. These cardiac biomarkers were disseminated into the bloodstream due to damage caused by free radicals that doxorubicin inflicted on cardiac myocytes (Abd-Allah et al., 2002; Renu et al., 2018).

A decrease in mortality rate and reduction in CK-MB, troponin I and LDH levels following administration of *J. mollissima* extract at 400 and 600 mg/kg were indicative of the cardioprotective activity of *J. mollissima*. Due to its phenols, carotenoids, and anthocyanins, J. mollissima may possess antioxidant properties (Manzoor et al., 2022). As a result of its free radical scavenging activity, *J. mollissima* protects against lipid peroxidation by dramatically lowering its concentration. The doxorubicin molecule binds strongly to the heart tissue phospholipids and cardiolipin. Rats that were administered doxorubicin exhibited elevated plasma sodium levels in comparison to controls; however, those that were administered J. mollissima experienced a return to normal levels. Rats who have suffered cardiac injury have also been found to have elevated sodium levels (Öz and İlhan, 2006).

Serum potassium levels were lower in the doxorubicin group than in the control group, consistent with earlier research indicating that cardiomyopathy can cause hypokalemia. J. mollissima therapy, on the other hand, restored the intoxicated group’s potassium levels to nearly normal, as demonstrated in previous studies (Iqbal et al., 2022).

In rats, doxorubicin decreased acetyl-CoA levels, slowed glucose synthesis, reduced urea generation, and inhibited lipid peroxidation (Ruggeri et al., 2018). At dosages of 400 and 600 mg/kg, *J. mollissima* significantly reduced oxidative stress induced by doxorubicin and improved the deterioration of heart function (p < 0.005), according to this study. The phenols in J. mollissima are potent antioxidants, so they may scavenge radicals and provide hydrogen. Antioxidants are a type of secondary metabolite that plants naturally contain (Ain et al., 2023).

Previous studies have demonstrated that gallic acid exhibits a cardioprotective effect through various mechanisms, including the inhibition of lipid peroxidation and platelet aggregation by directly inhibiting thrombin, the enhancement of antioxidant enzyme activity, and the reduction of cardiac biomarkers. Previous studies showed that gallic acid reduces CK-MB, CPK, cTnT, LDH, LDL, and triglycerides by inhibiting lipogenesis. Gallic acid is also used to treat ventricular hypertrophy. It can protect against vascular diseases by down-regulating the expression of angiotensin-II, NOX-2, and angiotensin-converting enzymes. It can also decrease vascular calcification by inhibiting the BMP-2 signalling pathway. Therefore, it was thought that the presence of gallic acid, as shown in the HPLC analysis of leaves of *J. mollissima,* also has cardioprotective effects. The results of this study revealed that it has anticoagulant, antioxidant, and thrombolytic properties (Akbari, 2020).

Previous studies have revealed that flavonoids, such as rutin and quercetin, have cardioprotective effects, as these metabolitess can decrease the risk of cerebrovascular and cardiovascular diseases by protecting vascular health. These metabolitess have anticoagulant activity by reducing fibrinogen levels and inhibiting platelet activity. Quercetin has been reported to inhibit the amidolytic activity of activated factors X and II. It was proposed that the β ring in flavonoids has -OH group structures that may inhibit the activity of factor Xa and thrombin (Sabbagh et al., 2023; Weremfo et al., 2011; Ogston and Douglas, 1971). Rutin is also considered to have anti-factor Xa activity and has been reported as a thrombolytic agent, as it can block the enzymatic activity of protein disulfide isomerase (PDI) (Dar and Tabassum, 2012; Sabbagh et al., 2023). Quercetin, rutin, and mandelic acid were considered major antioxidant constituents that decrease the oxidative stress caused by ROS. Previous studies revealed that under an alkaline environment, a chain reaction of self-oxidation of pyrogallol can increase the production of O_2_, which may prove beneficial in antioxidant activity (Nataraj et al., 2022). The presence of polyphenols, flavonoids (such as quercetin), and tannins in plants has been demonstrated in previous research to possess thrombolytic properties.

Aquous methanolic leaf extract of *J. mollissima* showed dose dependent vasodilatory effect when tested against spontaneous and K 80 induced contractions in an isolated aortic strip similar to the standandard vasodilator verapamil (Figure 13). Vasoconstriction, ischemia and necrosis are the primary toxicities caused by the doxorubicin (Manzoor et al., 2022), and the results of our study showed that J. mollissima dose dependently reverse the vasoconstriction that could help in reducing the cardiotoxicity profile of doxorubicin. Calcium channel leakage/over expression of voltage gated calcium channels is one of the major biochemical pathway of doxorubicin induced toxicity in cardiovascular system; ischemia, coagulation, necrosis, cardiac overload and ventricular hypertrophy (Imran et al., 2021; Imran et al., 2022, Imran et al., 2023). J. mollissima when tested for voltage gated calcium blocking activity against K 80 indced hypersecretion of Ca ions in aortic strip, J. mollissima dose dependedntly blocked voltage gated calcium channels in a similar fashion as standard calcium channel blocker verapamil (Figure 13). Furthermore, calcium channel response curves were constructed and tested with *J. mollissima* and verapamil. Similar pattern of response was recorded in dose response curves (Figure 14), which adds another cardioprotectivie feature to *J. mollissima.*

Cardiac inflammation plays a crucial role in the development of cognitive dysfunction in cardiac function, as insulin resistance causes systemic inflammation that extends to the brain (Rom et al., 2019). Additionally, the overactivation of microglia and astrocytes leads to the excessive production of proinflammatory cytokines, including TNF-α, IL-1β, and IL-6. In contrast, reducing anti-inflammatory cytokines, such as IL-10, further exacerbates the inflammatory response (Qin et al., 2023; Muehlberg et al., 2023; Kirstein, 2009; Markman et al., 2004). Therefore, we hypothesize that *J. mollissima*-mediated improvements in cognitive function may be linked to its ability to regulate cardiac inflammation. Our results indicated that J. mollissima treatment at 400 and 600 mg/kg significantly decreased TNF-α, IL-1β, and IL-6 levels while increasing IL-10 expression, thereby reducing cardiac inflammation.

This investigation demonstrated that J. mollissima leaf extract, in an aqueous-methanolic form, exhibits a notable increase in various coagulation parameters, including CT, PT, and APTT, in in vitro experiments, as well as BT *in vivo* experiments, when compared with heparin and distilled water. According to research, *J. mollissima* contains phenols, flavonoids, and flavone, all of which have antioxidant properties and can protect the heart from damage caused by doxorubicin.

The study has limitations, including the absence of detailed mechanistic pathway validation to fully elucidate how *J. mollissima* extracts confer their protective effects against doxorubicin-induced cardiotoxicity. The drug interaction between doxorubicin co-administration with *J. mollissima* extracts and effect on the efficacy of doxorubicin was not explored. Future research should focus on isolating the active phytoconstituents responsible for the cardioprotective effects and conducting in-depth mechanistic studies targeting specific biochemical and molecular pathways If validated through mechanistic studies and clinical trials, the cardioprotective effects of *J. mollissima* could be developed into adjunctive therapies for patients undergoing chemotherapy, potentially mitigating doxorubicin-induced cardiotoxicity with a natural, plant-based approach.

## 5 Conclusion and recommendations

The results of this study suggest that delivering *J. mollissima* extract simultaneously with doxorubicin may alter the activity of antioxidant enzymes, cardiac enzymes, and pathways that induce cardiac damage. In light of this, suggesting that *J. mollissima* extract mitigates Doxo-induced cardiotoxicity without reducing doxorubicin’s therapeutic effectiveness may be prudent. However, assessing the pertinent signalling pathway still requires much investigation. Preventing cardiotoxicity, avoiding additional drugs, and improving the patient’s quality of life could be achieved by using *J. mollissima* extract before or during therapy with doxorubicin in cancer patients.

## Data Availability

The original contributions presented in the study are included in the article/supplementary material, further inquiries can be directed to the corresponding authors.
